# Supplementary material for: Reversible chirality inversion of an AuAgx-cysteine coordination polymer by pH change
Source: Nat Commun. 2024 Mar 6;15:2042. doi: 10.1038/s41467-024-45935-3 (PMC10918179; doi:10.1038/s41467-024-45935-3)
Supplement: Supplementary file 1 — Supplementary Information [file 41467_2024_45935_MOESM1_ESM.pdf]

Supplementary information

Reversible chirality inversion of an $AuAg_x$-cysteine coordination polymer by pH change

Bing Ni*[1], Dustin Vivod[2], Jonathan Avaro[3], Haoyuan Qi[4], Dirk Zahn[2], Xun Wang[5], Helmut Cölfen*[1]

1. Physical Chemistry, University of Konstanz, Universitätsstrasse 10, 78457 Konstanz, Germany
2. Friedrich-Alexander-Universität Erlangen-Nürnberg (FAU), Department of Chemistry and Pharmacy, Chair for Theoretical Chemistry/Computer Chemistry Centre (CCC) Nägelsbachstrasse 25, 91058 Erlangen, Germany
3. Center for X-ray Analytics, Biomimetic Membranes and Textile, Empa, Swiss Federal Laboratories for Materials Science and Technology, Lerchenfeldstrasse 5, St. Gallen CH-9014, Switzerland
4. Faculty of Chemistry and Food Chemistry & Center for Advancing Electronics Dresden (cfaed), Technische Universität Dresden, 01062 Dresden, Germany
5. Key Lab of Organic Optoelectronics and Molecular Engineering, Department of Chemistry, Tsinghua University, Beijing, 100084 China

Deceased: Helmut Cölfen

Email: Bing Ni; Helmut Cölfen, bing.ni@uni-konstanz.de; helmut.coelfen@uni-konstanz.de;

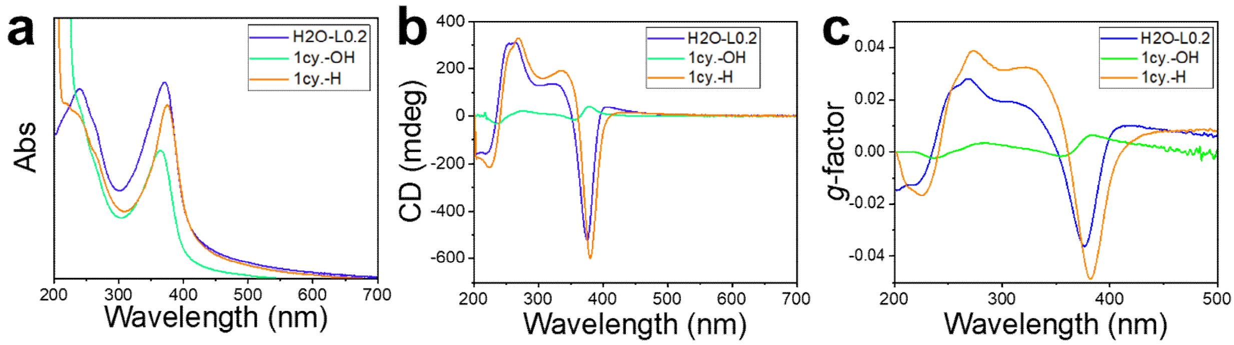

Supplementary Figure 1 Responsive behavior of the coordination polymer (CP) material prepared in H_2O.
(a, b, c) UV-Vis spectra, CD spectra, and *g*-factors of the original L0.2 (blue lines), and the L0.2 after 1 cycle of alkalization (1cy.-OH, green lines) and acidification (1cy.-H, orange lines) process. 0.2 denotes the Ag/Au ratio. Source data are provided as a Source Data file.

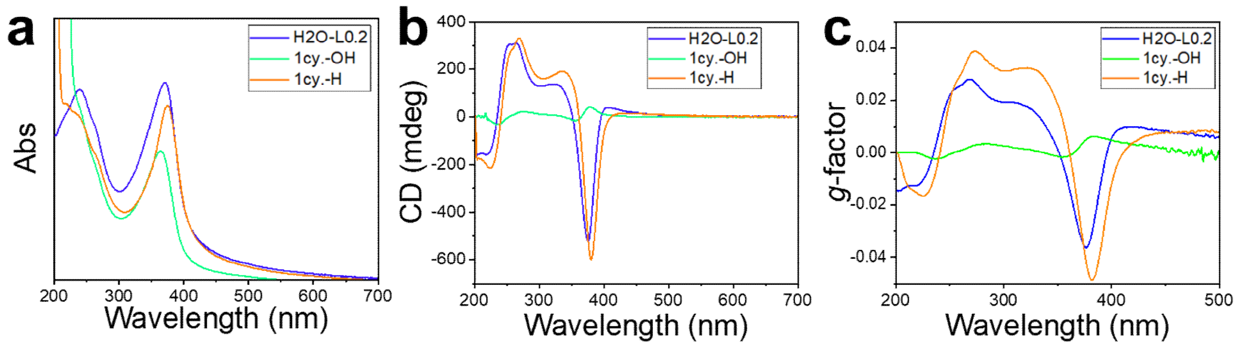

Supplementary Figure 2 Responsive behavior of the CP material prepared in a CTAC solution.
(a, b, c) UV-Vis spectra, CD spectra, and *g*-factors of the original L0.06 (blue lines), and the L0.06 after 1 cycle of alkalization (1cy.-OH, green lines) and acidification (1cy.-H, orange lines) process. 0.06 denotes the Ag/Au ratio. The measured RE was 67% in the first alkalization process. Source data are provided as a Source Data file.

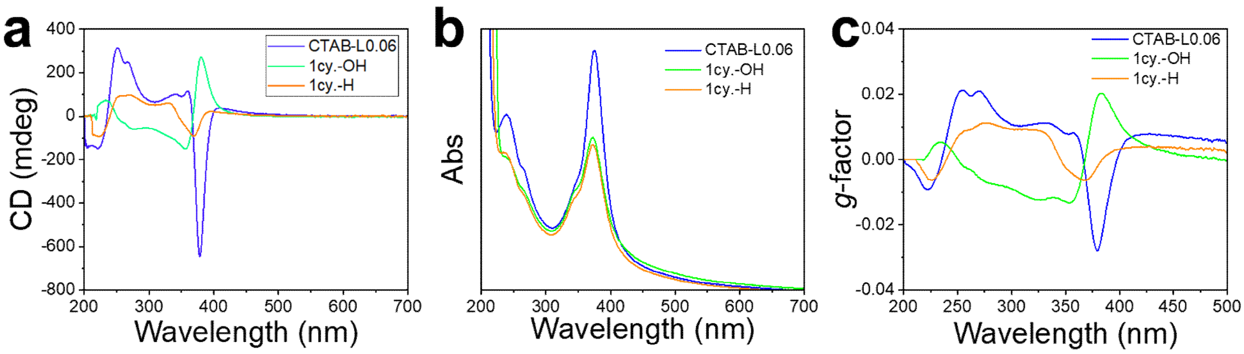

Supplementary Figure 3 Responsive behavior of the CP material prepared in a CTAB solution.
(a, b, c) UV-Vis spectra, CD spectra, and *g*-factors of the original L0.06 (blue lines), and the L0.06 after 1 cycle of alkalization (1cy.-OH, green lines) and acidification (1cy.-H, orange lines) process. 0.06 denotes the Ag/Au ratio. The measured RE was 80% in the first alkalization process. However, the acidification process cannot largely recover the original *g*-factor plots. Source data are provided as a Source Data file.

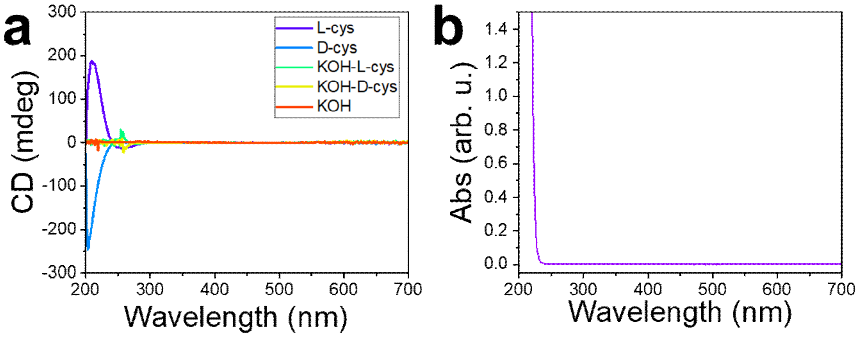

Supplementary Figure 4 Optical properties of cysteine and KOH solutions.

(a) CD spectra of cysteine and cysteine-KOH solution; (b) UV-Vis spectrum of 1M KOH solution.

Due to the strong absorbance of KOH solutions below 230nm, mainly noises were detected at the CD spectra below 230 nm in the existence of KOH. Thus, the optical activities of KOH-L-cys and KOH-D-cys cannot be clearly measured by the CD spectrometer. Source data are provided as a Source Data file.

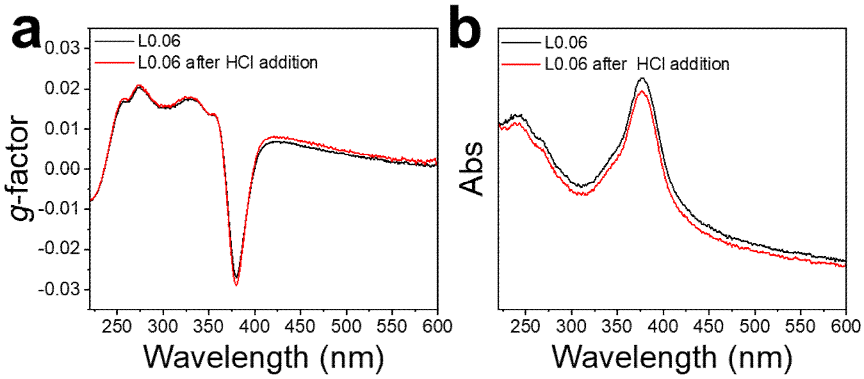

Supplementary Figure 5 Responsive behavior of the CP material prepared in a CTAB-decanol solution. (a) CD spectra and (b) UV-Vis spectra of the original L0.06 (back lines) and the L0.06 after HCl addition (red lines). After the HCl addition, the concentration of HCl in the final solution is 1 M. Source data are provided as a Source Data file.

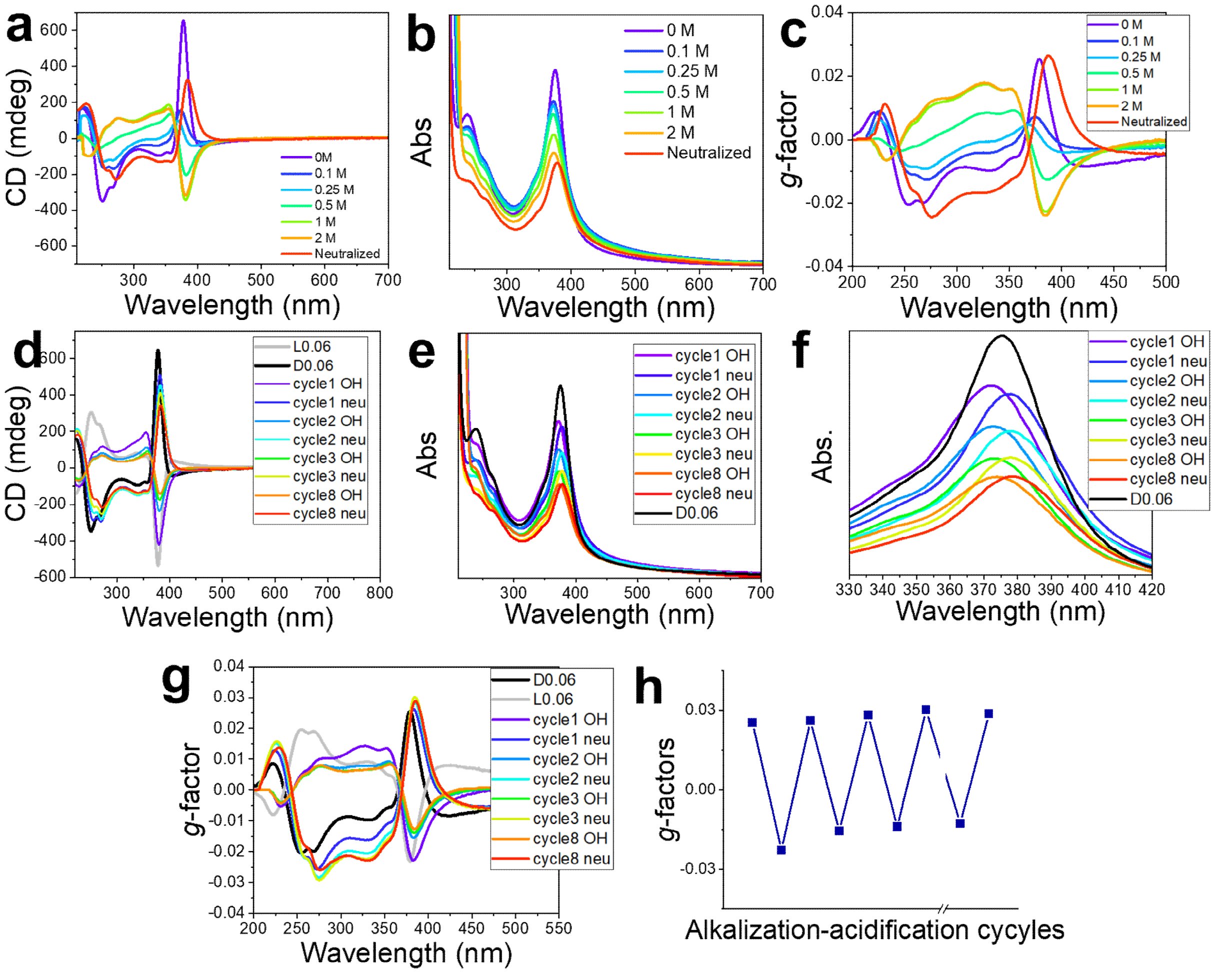

Supplementary Figure 6 The responsive behavior of D0.06

(a-c) Optical property changes along with the increase of KOH concentration. The solution was neutralized by HCl at the end;

(d-g) Optical property changes of D0.06 upon alkalizations and acidifications. In the figure legend, alkalization process is marked by “OH”, and neutralization is marked by “neu”. The spectrum of L0.06 is added here as reference (the grey line). (f) shows an enlarged area in (e). (h) shows the changes of the main *g*-factor peak of the Aurols.

Source data are provided as a Source Data file.

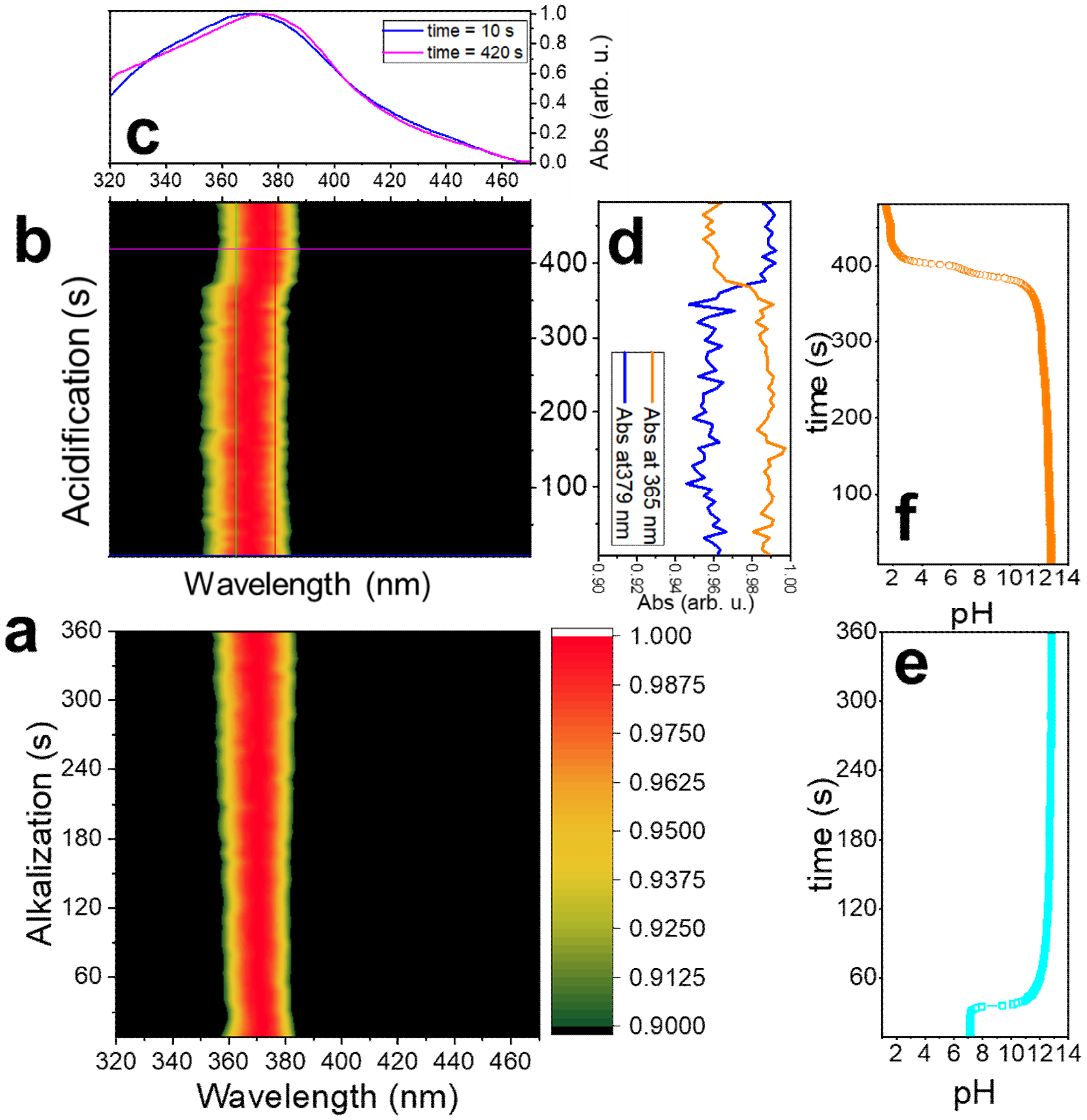

Supplementary Figure 7 UV titration results of L0.06.

The UV spectra were normalized and only intensities between 0.9 and 1.0 were shown to clearly illustrate the peak center change here. The UV peak center blue-shifts and gradually broadens during alkalization (a), and suddenly red-shifts during acidification (b). (c) shows the typical UV-Vis spectra before and after the sudden red-shift during the acidification. (d) shows the relative absorption at 365nm and 379nm during the whole acidification process. The pH changes during the titration were simultaneously recorded with a connected pH electrode (e, f).

Source data are provided as a Source Data file.

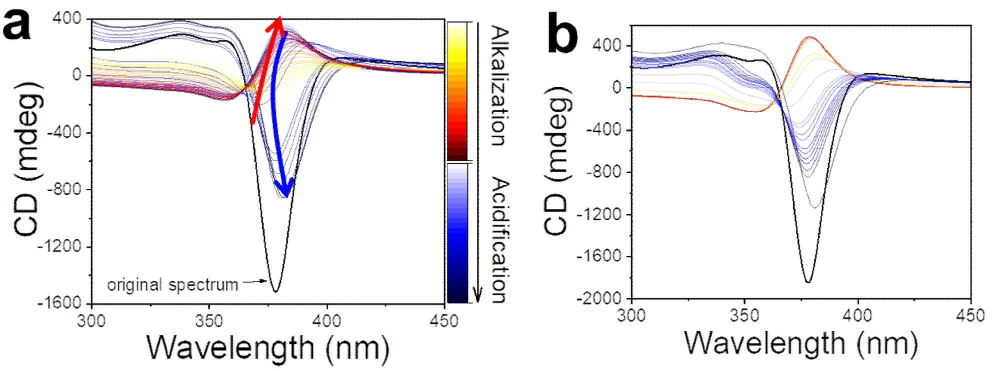

Supplementary Figure 8 CD titration results of L0.06.

(a) titration in H_2O; (b) titration in a phosphate buffer solution (0.01M) to slow down the pH changes at the corresponding titration point. Highly concentrated KOH solution (11M) and HCl solution (≈12M) were used to achieve alkalization and acidification. The pHs were recorded by a separated pH meter (see details in the experimental part). The ratio of the acid form is estimated by the relative intensity of the main peaks. The CD_{379nm} intensity after the acidification process is solely caused by the acid form, and the CD_{365nm} intensity after the alkalization process is solely caused by the alkali form. The intermediate intensity is the linear combination of the two forms. Then the ratio of acid form at different pHs can be roughly calculated and shown at Figure 2b. The value slightly larger than 1 might be caused by the concentration change during the titration. The change of the acid form ratio estimated according to (b) overlapped well with those obtained from (a) (Figure 2b), suggesting that the buffer solution would not interfere with the transitions and that the transition process was gradual. Source data are provided as a Source Data file.

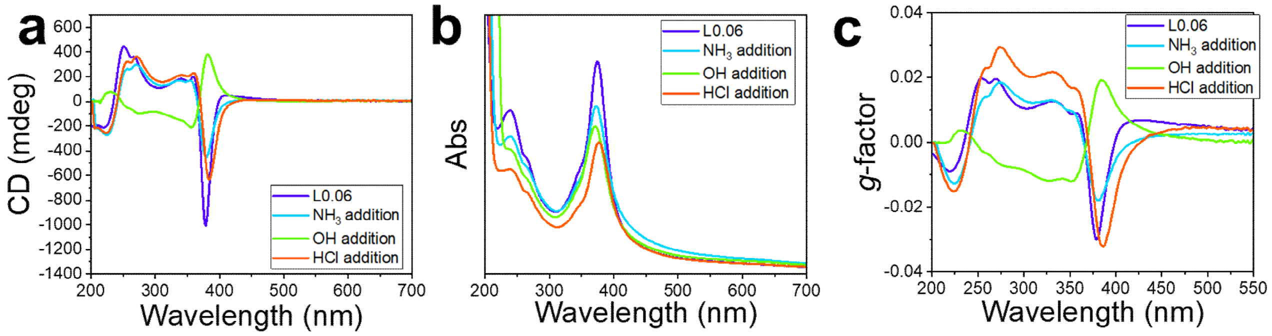

Supplementary Figure 9 Further alkalizing the NH_3 solution added L0.06 CP solutions.

(a) CD spectra; (b) UV-Vis spectra; (c) *g*-factor plots. Blue lines mark the original spectra of L0.06, cyan lines mark the NH_3 solution added L0.06 CP solutions. Further KOH addition could lead to the inversion of the chirality (green lines), and the neutralization of the solution by HCl (red lines) leads to the recovery of the original optical activity. Source data are provided as a Source Data file.

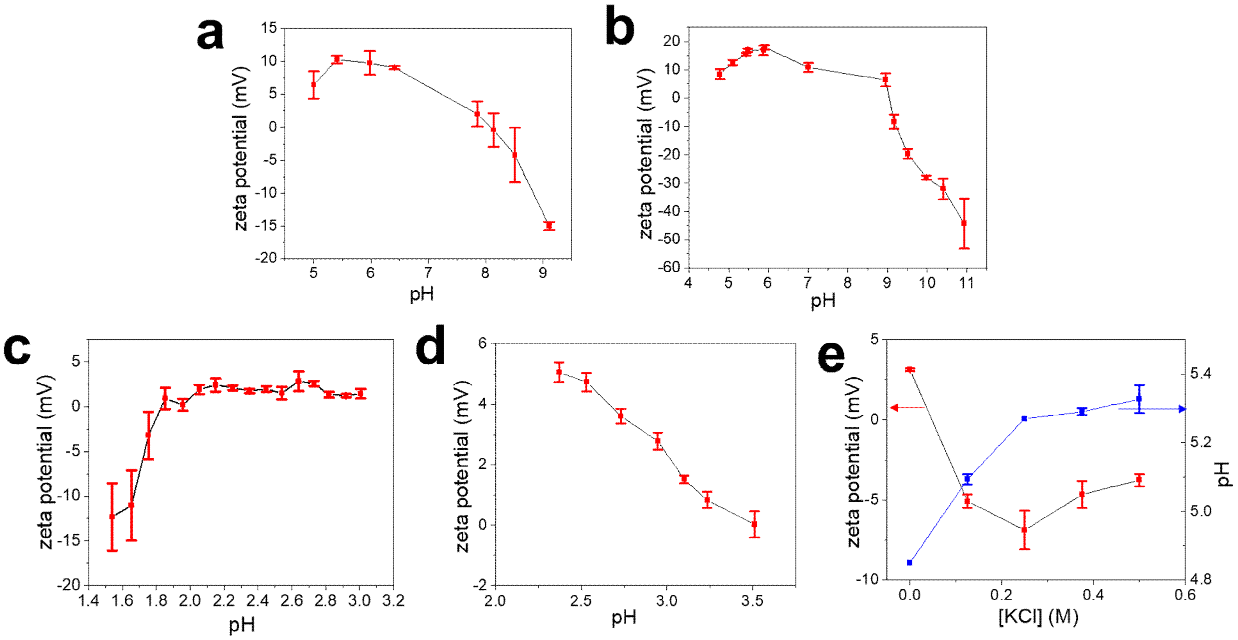

Supplementary Figure 10 Zeta potential titrations of L0.06 with different titrants.

(a) KOH; (b) NH_3; (c) HCl; (d) H_2SO_4; (e) KCl, the red line indicates the zeta potential changes, the blue line marks the pH changes, during the KCl solution titration. Error bars indicate the standard deviation between 3 consecutive measurements.

Normally, the zeta potential would increase as the pH decreases due to protonation (d). However, the titration in HCl (c) showed a decrease. The reason could be attributed to the binding of Cl^-. Furthermore, (e) directly demonstrates that the coordination polymer can bind Cl^-, leading to the unusual decrease in zeta potential upon acidification.

Source data are provided as a Source Data file.

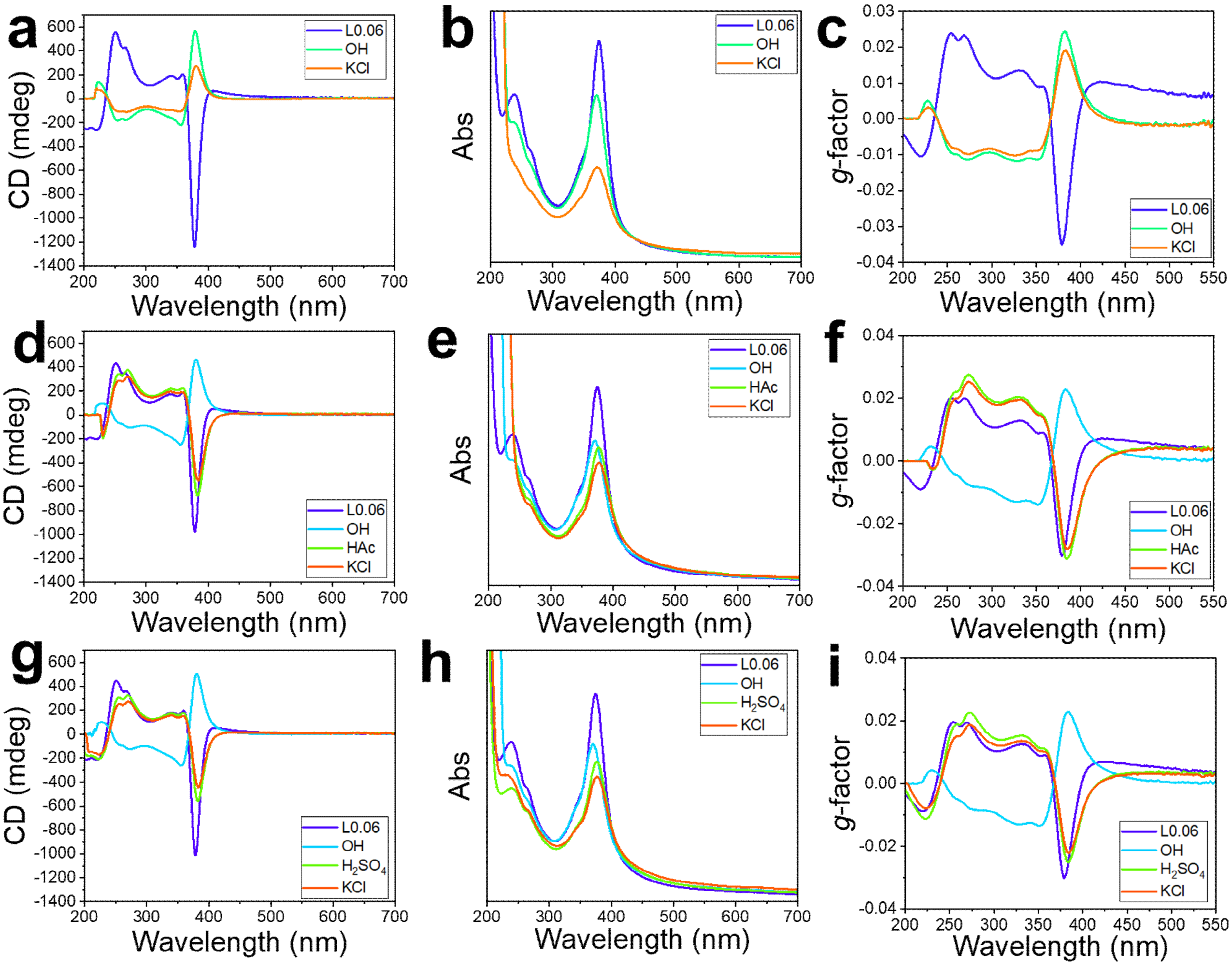

Supplementary Figure 11 Inducing chirality responsive behavior of L0.06 using other ions.

The results demonstrate that K^+, Cl^-, CH_3COO^- (Ac^-), or SO_4^{2-} do not induce the responsive behavior. L0.06 represents the original spectra, and the top-to-bottom order in the legend indicates the sequence of extra solution addition.

(a-c) The addition of OH^- inverts the optical activity to the alkalization state. Subsequent addition of KCl fails to invert it back (not like HCl), resulting in the structure remaining in the alkalization state.

(d-f) The addition of OH^- inverts the optical activity to the alkalization state. Then, HAc is added to invert it back to the acidification state. If Ac^- is capable of inverting the structures as well, the structure would remain in the alkalization state because Ac- is the remaining ion after the reaction between H^+ and OH^-. However, KCl following the addition of Ac^- fails to invert it back to the alkalization state, resulting in the structure staying in the acidification state. Since KCl has no influence on the alkalization state (a-c) and the acidification state (d-f), K^+ and Cl^- have no influence on the responsive behavior.

(g-i) Performing the same analysis with SO_4^{2-} reveals that SO_4^{2-} is unable to invert the structures.

Source data are provided as a Source Data file.

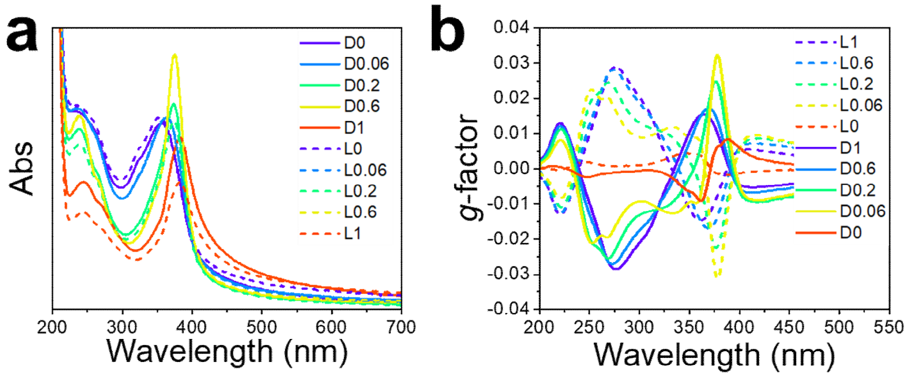

Supplementary Figure 12 UV-Vis spectra and *g*-factors of AuAg*x*-cys coordination polymers prepared in CTAB-decanol solutions with varying amounts of Ag doping.

The numbers in the Supplementary Figure legends represent the molar ratio of Ag to Au.

In (a), the AuroIs of D0 exhibited a broad peak ranging from ≈300 nm to ≈400 nm. The main peak appeared around 355 nm, accompanied by a shoulder peak at approximately 320 nm. As the amount of Ag increased, the main peak gradually red-shifted to 382 nm for D1, while the shoulder peak became less prominent. This peak is indicative of the presence of aurophilic interactions[1,2].

In (b), the optical activities underwent more significant changes with increasing amounts of Ag. For AuAgx-D-cys materials, the *g*-factors showed negative values from ≈230 nm to ≈350 nm, and exhibited a positive peak from ≈350 nm to ≈400 nm. The positive peak gradually blue-shifted from 388 nm to 360 nm with increasing Ag doping, and its intensity initially increased and then decreased. This peak likely arises from a geometric structure that chiral-couples the S-Au bond localized ligand-metal charge transfer transitions via aurophilic interactions[3,4]. The negative part of the spectrum exhibited increased intensity as the Ag doping increased.

Source data are provided as a Source Data file.

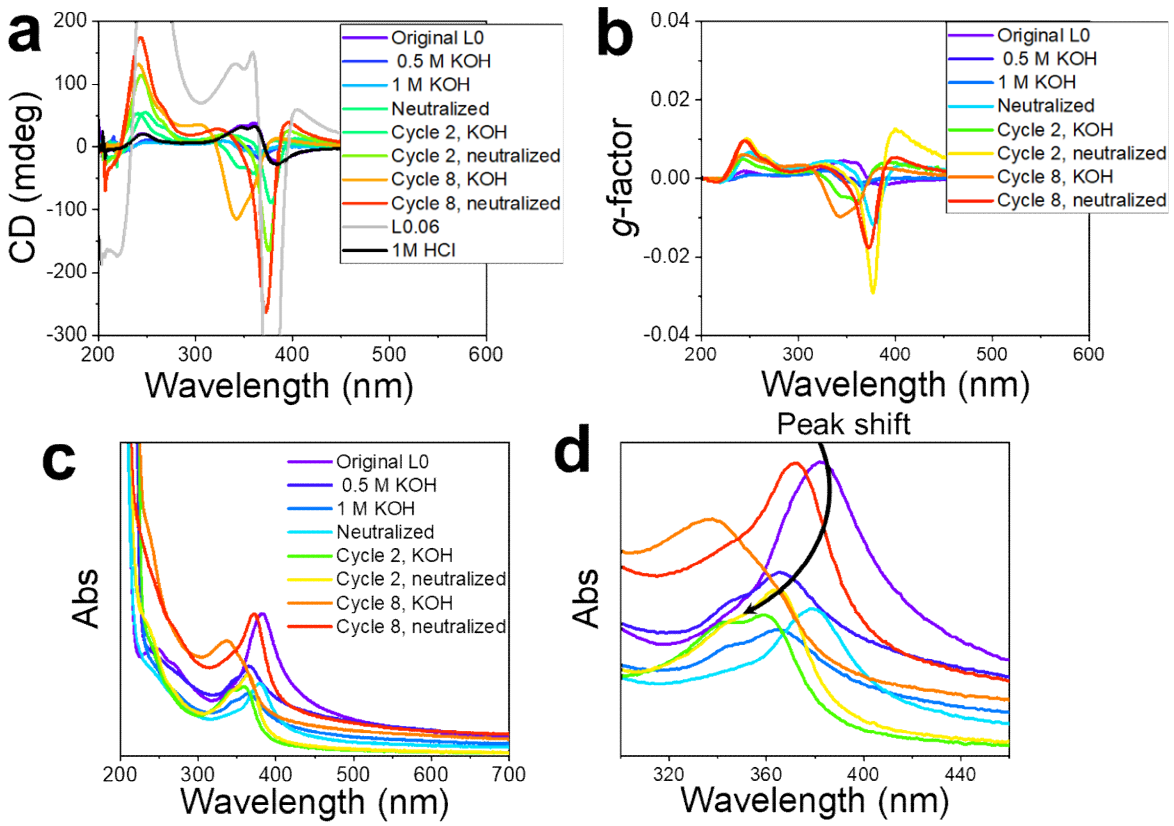

Supplementary Figure 13 Responsive behavior of L0 in different pHs.

(a) CD spectra; (b) *g*-factor plots; (c, d) UV-Vis and enlarged UV-vis spectra. The graphs share the same figure legend. The neutralization was checked by pH paper. No obvious inversion was found. Source data are provided as a Source Data file.

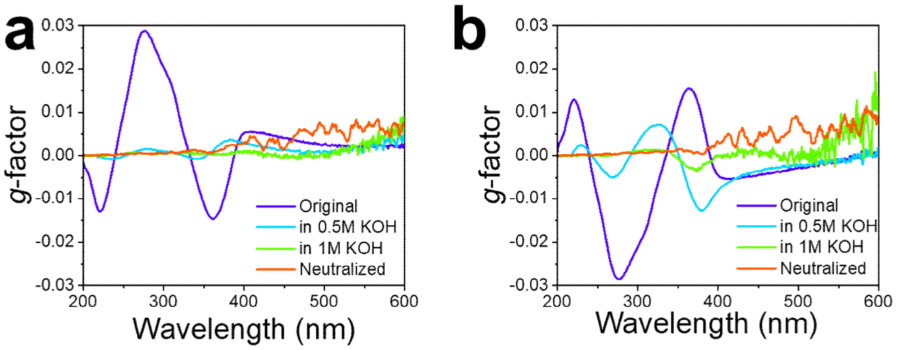

Supplementary Figure 14 Irreversibility of CP materials with higher Ag contents.

(a) *g*-factor plots of L0.6; (b) *g*-factor plots of D0.6. The graphs showed low stability when the Ag^{+} was large. Source data are provided as a Source Data file.

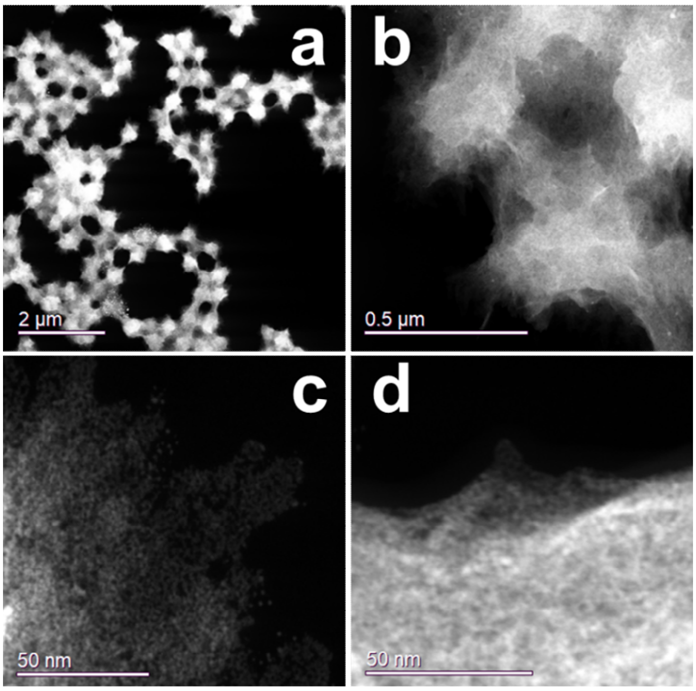

Supplementary Figure 15 STEM images of the L0.
(a-d) STEM images with different magnifications.

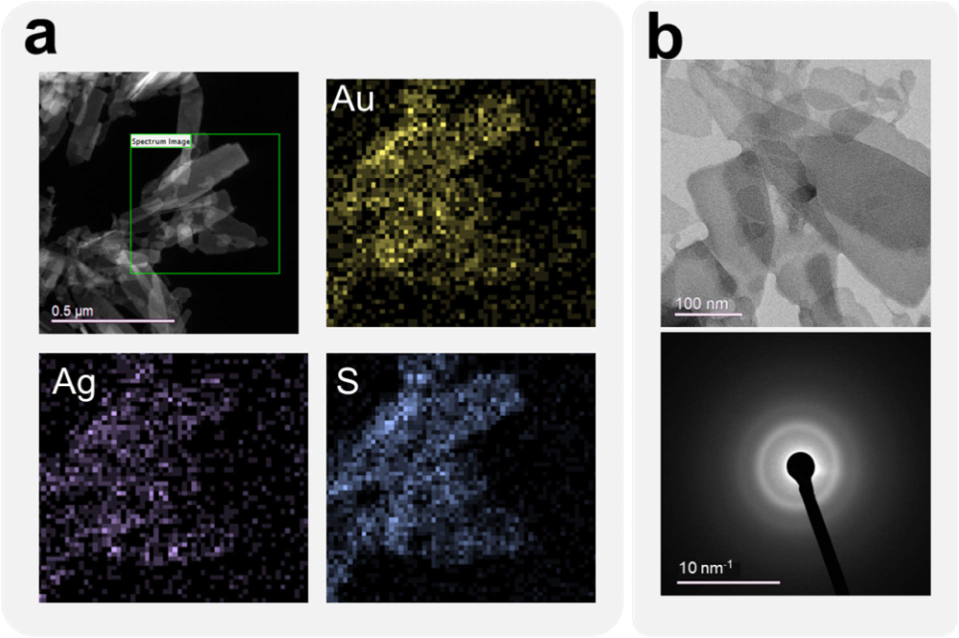

Supplementary Figure 16 Further TEM characterizations of L0.06.
(a) EDX mapping and (b) SAED of petals of L0.06

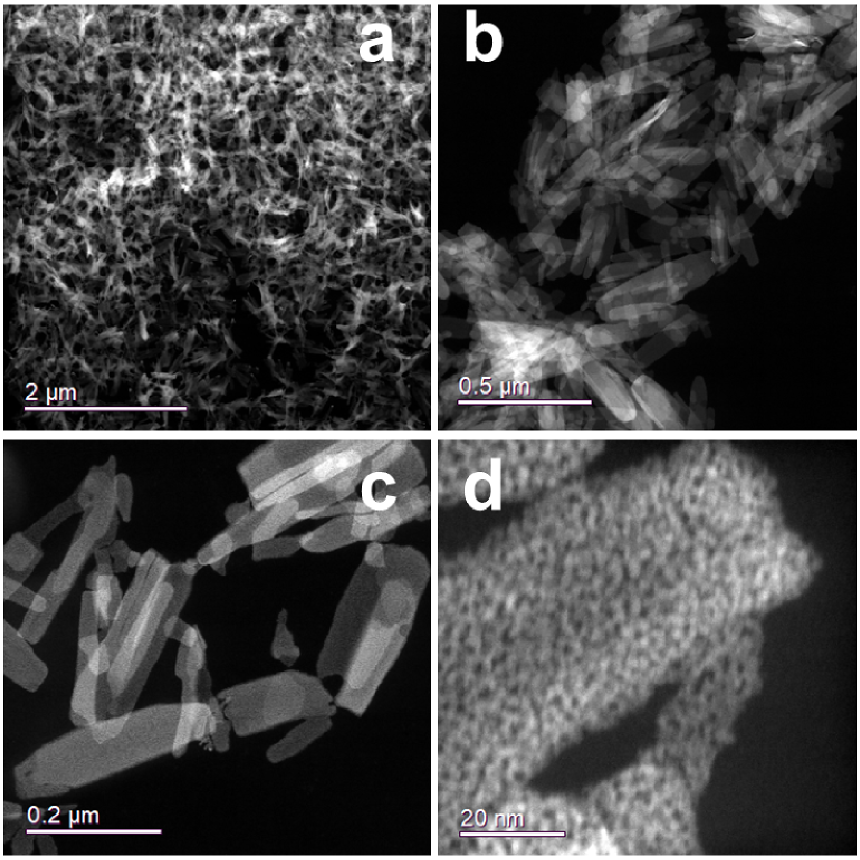

Supplementary Figure 17 STEM images of L1
(a-d) STEM images with different magnifications.

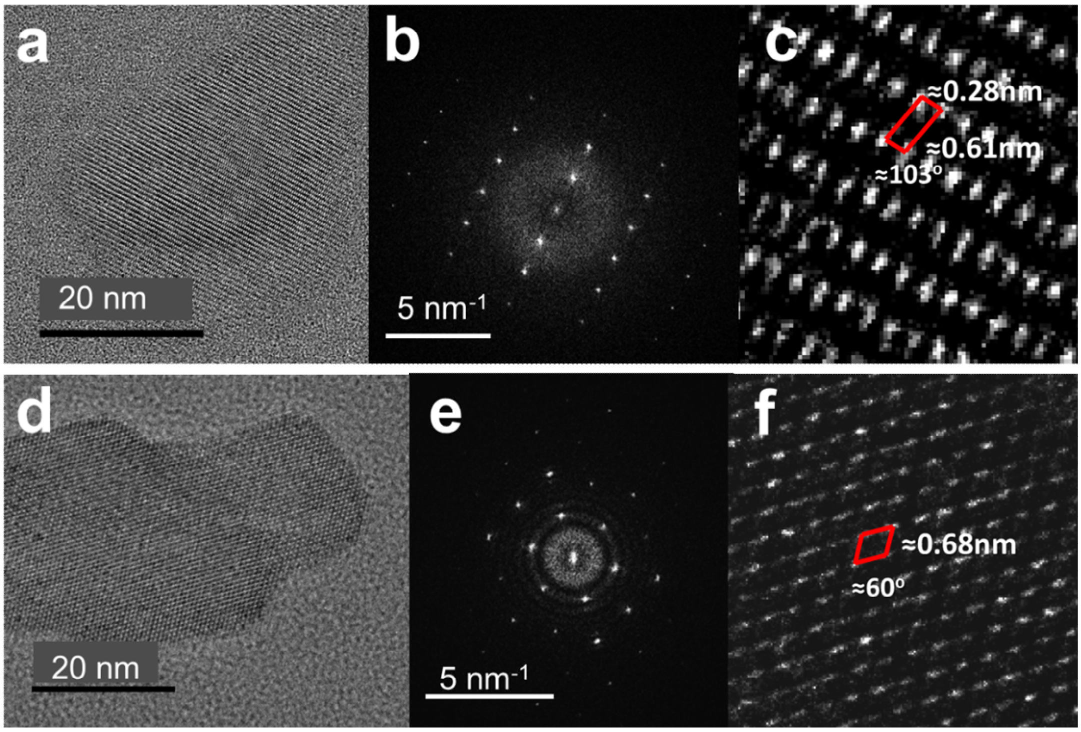

Supplementary Figure 18. HRTEM characterization of D0.06.
Some HRTEM images of the D0.06 showed lattice fringe (a, d). (b, e) show the FFT patterns. (c, f) enlarged HRTEM images, respectively.

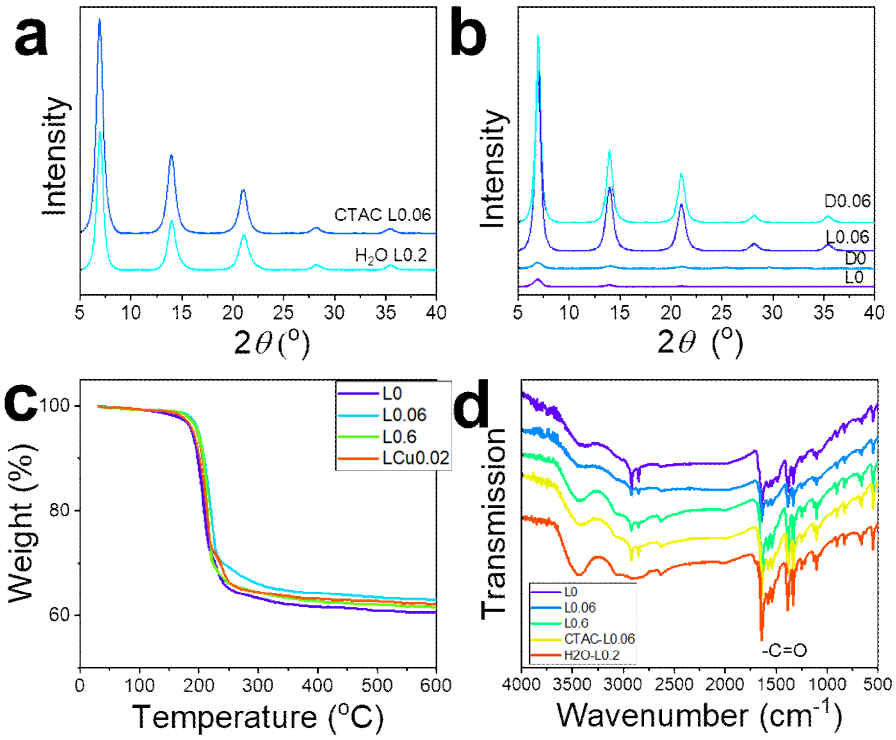

Supplementary Figure 19 Further characterizations of CP materials with different Ag content or metal doping. (a, b) XRD results of the coordination polymers prepared at H_2O, CTAC (a) and CTAB-decanol (b) solutions. The group of peaks with proportional 2θ indicate a layered structure, and the spacing is 1.2nm according to the Bragg equation ($2d\sin\theta=n\lambda$, with different n). The inexistence of other peaks suggested the non-crystalline feature of the individual layers. (c) TGA and (d) FT-IR results. Source data are provided as a Source Data file.

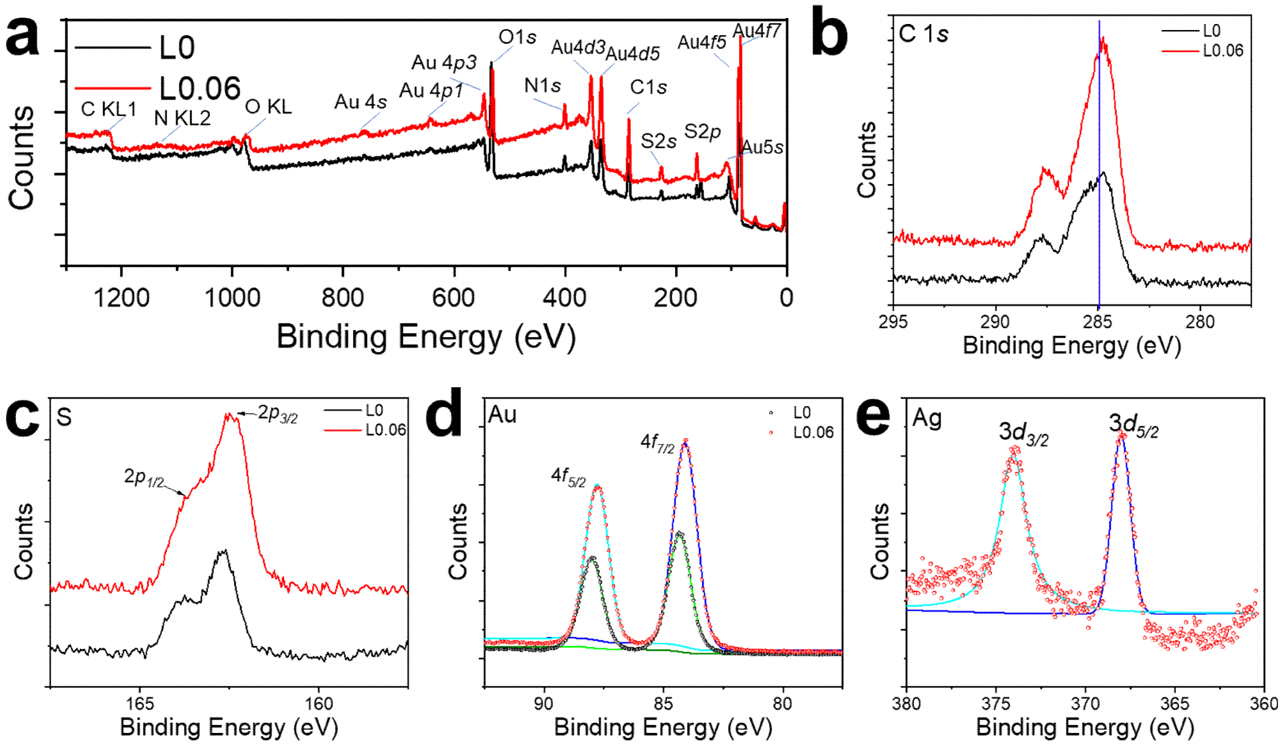

Supplementary Figure 20. XPS spectra of the L0 and L0.06

The atomic percentages of different elements are shown in Supplementary Table 2.

(a) Survey of the full spectra of L0 and L0.06

(b) C 1*s* high-resolution scans. The peaks with the highest intensity in L0 and L0.06 are centered at the same binding energy (284.75 eV). Therefore, no calibration was applied to the spectra.

(c) S 2*p* high-resolution scans. The reported -SH and disulfide bonds in the literature exhibit a wide range of binding energies (162 eV to 165.1 eV)[5] [6,7] [8], and sometimes overlapping[9]. The peak centers of $2p_{1/2}$ and $2p_{3/2}$ are quite close (the labeled "$2p_{1/2}$" peak is not the shakeup signal of the strong $2p_{3/2}$ peak), which makes the peak differentiation and binding energy identification difficult. Furthermore, the binding energies are sensitive to the degree of protonation (protonation would shift the binding energy to higher energies[10]), which makes the differentiation of disulfide bonds further difficult.

(d, e) Au 4*f* and Ag 3*d* high-resolution scans. The peaks exhibit symmetry, eliminating the need for deconvolution processes. The binding energies of Au ($4f_{7/2}$ L0: 84.35eV; L0.06: 84.05eV) are higher than those of the typical metallic Au (83.95 eV[11]) and lower than the $HAuCl_4$ (85.70 eV[3]), and close to those reported Au(Ag)-cysteine coordination polymers[3,4]. For Ag, the $3d_{5/2}$ binding energies of metallic Ag, Ag_2O and AgO are quite close and all around 368eV. Here our Ag $3d_{5/2}$ binding energy is 367.90eV, close to that reported in Ag-doped Au-cys material[2,12].

Source data are provided as a Source Data file.

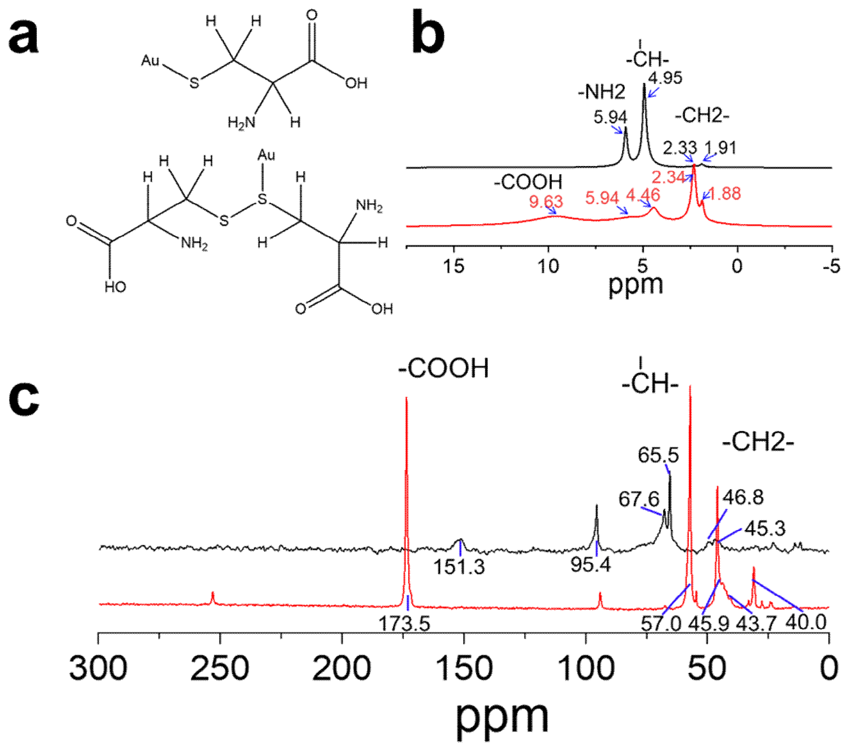

Supplementary Figure 21 Solid-state NMR results of L0 (black lines) and L0.06 (red lines)

The solid-state NMR spectra were measured by a JNM-ECZ600R spectrometer. The L0 and L0.06 were measured in the exact same method. For ^{13}CNMR, the resonance frequency was 150MHz. The tube diameter was 3.2mm. The test method was cpmas. The mas frequency was 12kHz. The relaxation delay was 3s. The accumulation of scans was 1200 times. The contact time was 2ms.

(a) shows the structure of Au-cys and Au-cystine structure

(b) shows the solid-state ^{1}HNMR spectra. It can be observed that the peaks of L0 and L0.06 are centered at similar positions, while L0.06 displays an additional broad peak at around 9.63 ppm, corresponding to -COOH. The ^{1}HNMR spectra cannot differentiate between cysteine and cystine, as the disulfide bond does not have protons.

(c) shows the solid-state ^{13}CNMR spectra. L0.06 exhibits intense peaks, while the peaks of L0 are weak. The peaks centered at 151.3 ppm (L0) and 173.5 ppm (L0.06) are attributed to -COOH. The two symmetric peaks of L0.06 (253 ppm and 93.0 ppm) around the -COOH peak are the mas sidebands resulting from the measurement technique and do not indicate ^{13}C signals. The peak centered at 95.4 ppm in L0 cannot be indexed, as typically only alkynes display peaks at such chemical shifts. This peak may arise due to the special chemical environment in the structure, as the ^{13}CNMR spectrum is highly sensitive to stereochemistry. However, we cannot directly deduce the structures based on these spectra.

Source data are provided as a Source Data file.

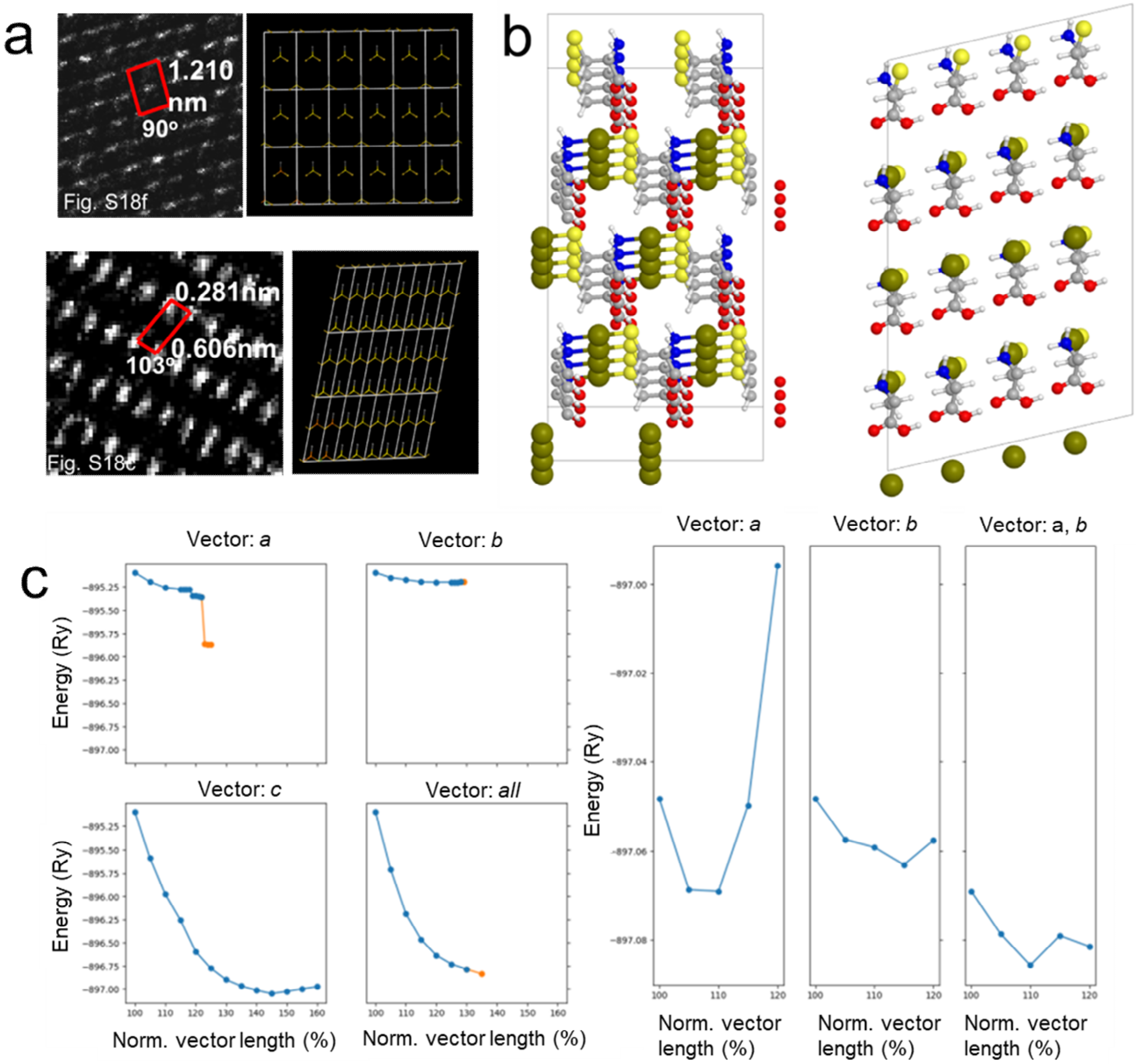

Supplementary Figure 22 DFT calculation of the Au-cys structure.

(a) Lattice fringes obtained from the TEM images. The distance of 0.281nm in is attributed to the aurophilic interactions. A body-centered/face-centered triclinic lattice with a=6.655Å, b=12.100 Å, c=2.810 Å, α=77°, β=90°, γ=90° could be constructed accordingly. It is worth mentioning that this lattice is not the unit cell of the structure, the lattice was just constructed to reflect the fringes shown here. (b) Optimized Au-cys structure. The Au-S distance and Au-N distance are 2.27 Å and 2.16 Å, respectively. Brown: Au; Cyan: Ag; Red: O; Yellow: S; Blue: N; Grey: C; White: H. (c) Change in energy in relation to changes in box vector magnitudes. Left: Initial cell relaxations from TEM structures. Right: Subsequent change after changing vector c by 145%. Orange points denote structures, which fragmented or where the gold structure reorganized.

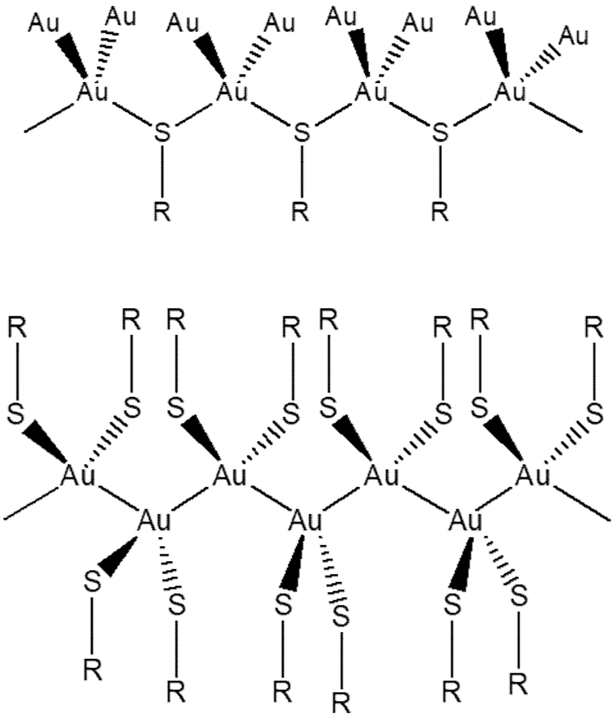

Supplementary Figure 23 Ideal structures of the Au-cys coordination polymers.

The upper panel shows the –Au-S- covalent chains, and the –Au-Au- aurophilic interactions in another dimension are not shown;

The lower panel shows the –Au-Au- aurophilic interactions, and the –Au-S- covalent chains in another dimension are not shown.

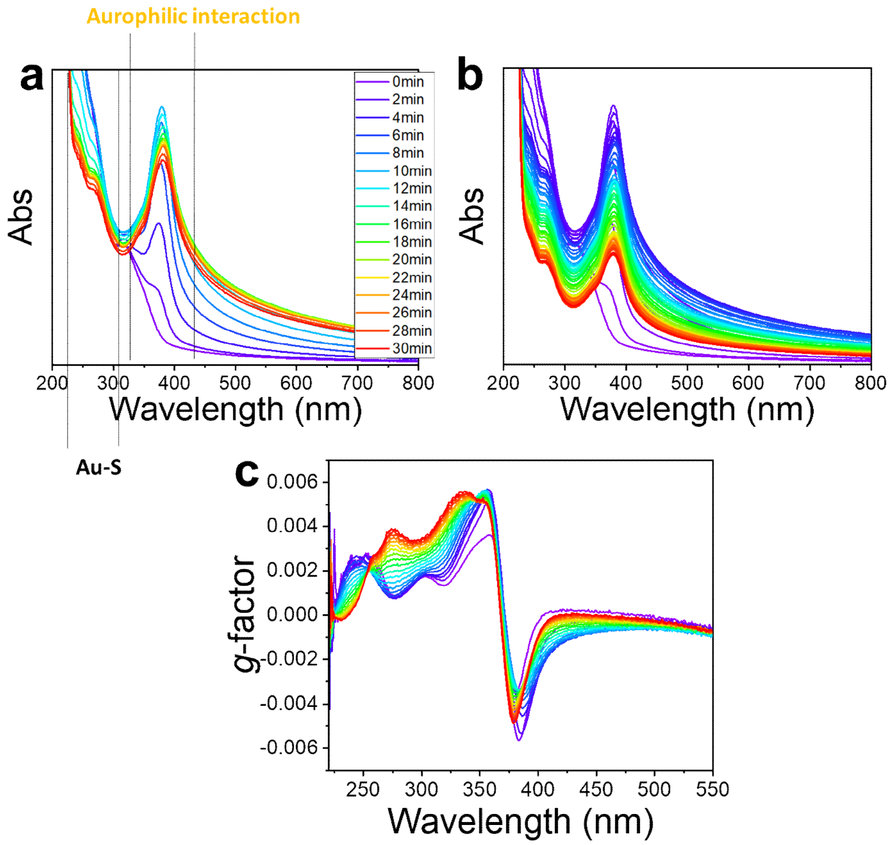

Supplementary Figure 24 Evolution spectra of the L0.06**.**

(a, b) *in-situ* UV-Vis spectrum to track the formation of L0.06. (a) initial 30min; (b) whole spectra evolution in 2 hours. There are two absorption peaks associated with the Au-S bond linear structures (<250nm, ≈270nm), and Au-Au.Ag aurophilic interactions assisted sheet structures (≈350nm, ≈378nm)[1], which are consistent with the reported results. The spectra of the first 10 min suggested the formation of Au-S bonds and the Au-Au/Ag aurophilic interactions. Then the shapes of the spectra would not change significantly, and only the intensity decreased due to the precipitation of the coordination polymers.

(c) The *g*-factor plots were calculated according to the in-situ CD spectra. The time interval here was 3 min due to the scan speed limit of the CD spectrometer, and the total tracking time here was 1 hour. It can be found that the intensities below 250 nm were decreasing, making the peak into a valley upon material growth, and the intensities around 250 nm ≈350 nm were increasing, making the valley into a peak. These suggested that the confirmation of the CP was continuously changing during the growth, even though the bonds (Au-S and Aurols) have been formed.

Source data are provided as a Source Data file.

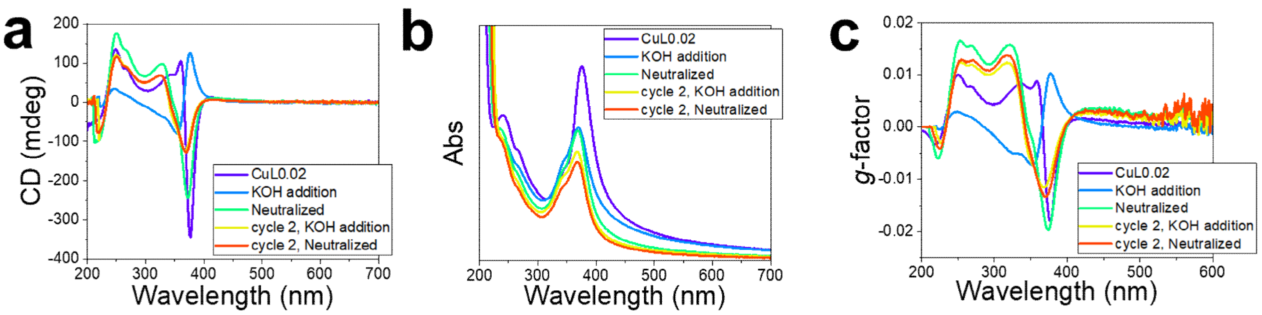

Supplementary Figure 25 Application of Cu cations to replace Ag cations in the CP materials.
(a) CD spectra; (b) UV-Vis spectra; (c) *g*-factor plots; to show the responsive behaviors of the obtained Cu doped CP materials under alkalization (KOH addition) and acidification (Neutralized) processes. Two alkalization-acidification cycles were tested. Source data are provided as a Source Data file.

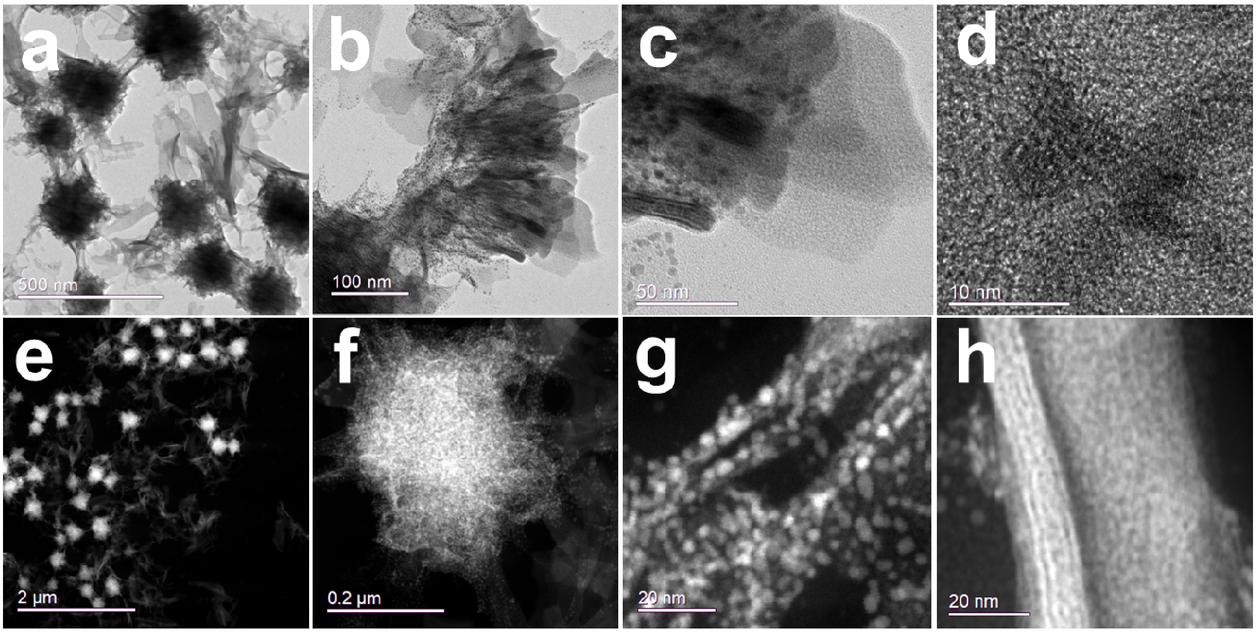

Supplementary Figure 26 TEM and STEM results of the Cu-L0.02 coordination polymers.
(a-d) TEM images at different magnifications.
(e-h) STEM images at different magnifications.

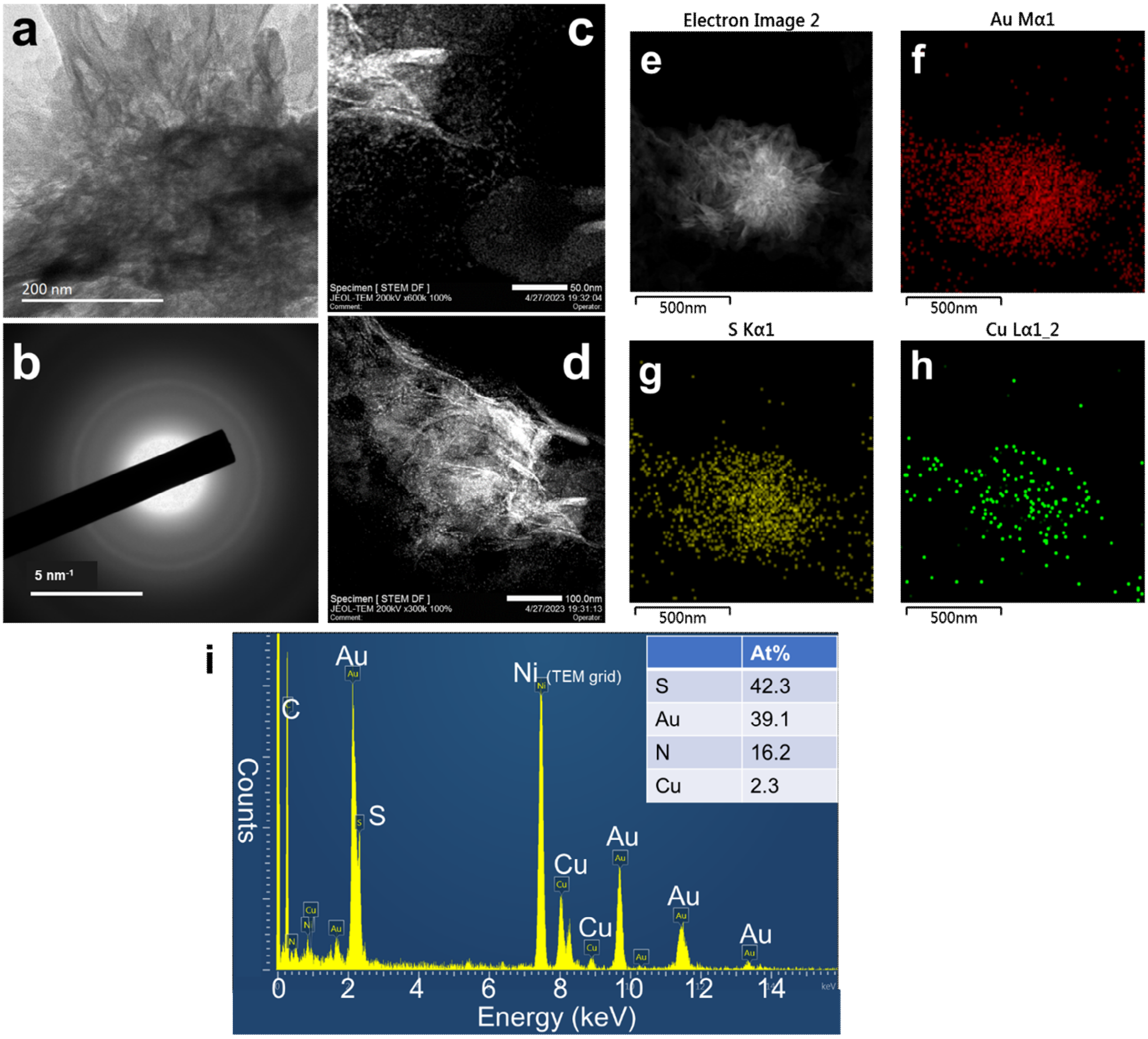

Supplementary Figure 27. Further structural characterization of the Cu-L0.02

(a, b) HRTEM image and the corresponding SAED patterns. The patterns suggested amorphous nature of the product.

(c, d) STEM images showing the layered structures and the small dots around the layers.

(e - h) EDX mapping of the Cu-L0.02 particles.

(i) EDX spectra of the Cu-L0.02 particles

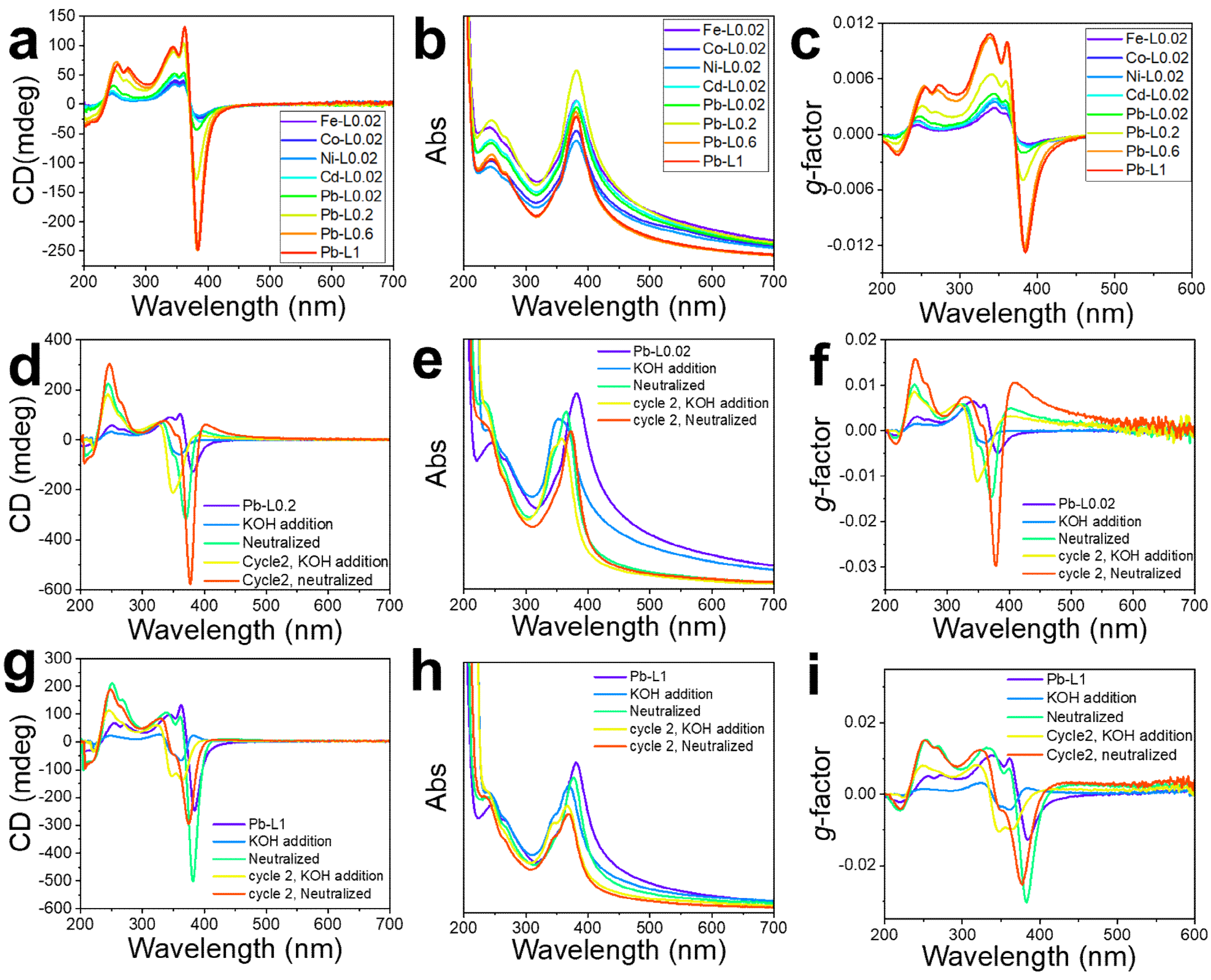

Supplementary Figure 28 The response of CP materials prepared with other metal doping.

(a-c) CD spectra, absorption spectra, and *g*-factor plots of Fe^{3+}, Co^{2+}, Ni^{2+}, Cd^{2+}, or Pb^{2+} doped Au-cys CP materials. Among all of them, Pb^{2+} (red lines in a-c) showed larger *g*-factors compared with those of the rest and the L0, which might be due to the weak interaction with Au(I). (d-f) Responsive behaviors of Pb-L0.02; (g-i) Responsive behaviors of Pb-L1.

Source data are provided as a Source Data file.

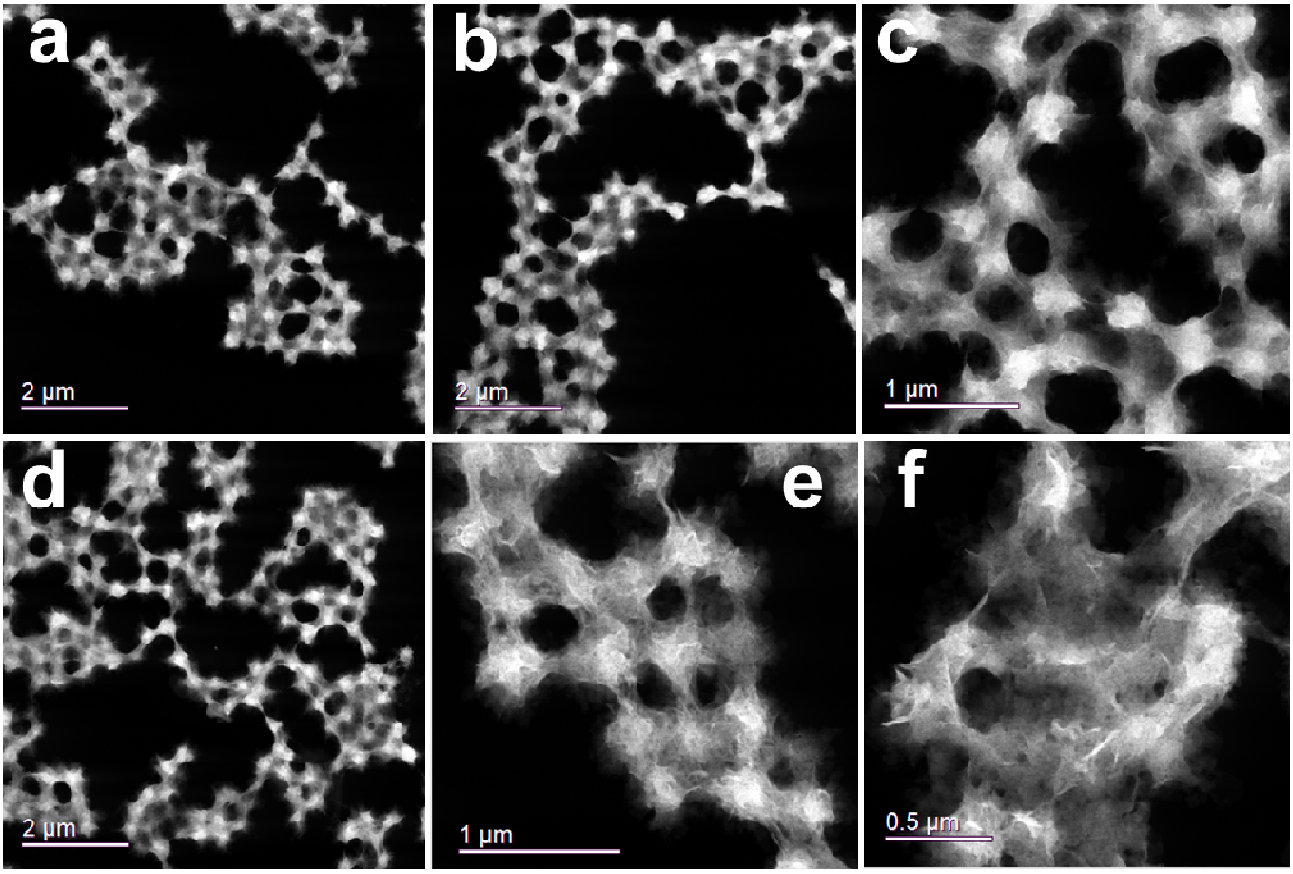

Supplementary Figure 29 STEM images of doped Au-cys CP materials.
(a) Fe-L0.02; (b) Co-L0.02; (c) Ni-L0.02, (d) Cd-L0.02, (e) Pb-L0.02, (f) Pb-L0.2.

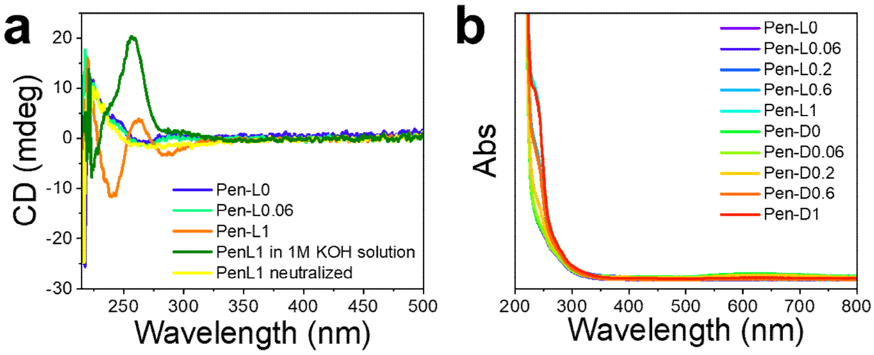

Supplementary Figure 30 Properties of the Au-Pen CP materials.

(a) CD spectra of Pen-L0, Pen-L0.06, and the responsive behavior of Pen-L1. The numbers denote atomic ratio of Ag:Au. For example, L0.06 means a Ag:Au ratio of 0.06:1. (b) UV-Vis spectra of different Au-Pen CP materials. Source data are provided as a Source Data file.

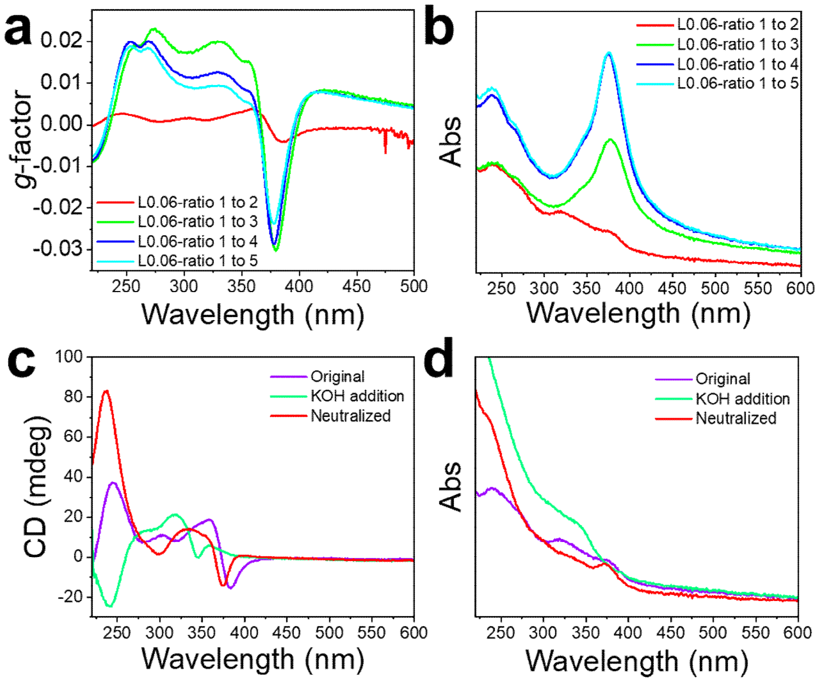

Supplementary Figure 31 For L0.06, changing the ratio of $HAuCl_4$ to L-cysteine could significantly tune the optical properties.

(a, b) *g*-factor plots and the absorption spectra;

(c, d) the responsive behavior of the products obtained with a $HAuCl_4$ to L-cystine ratio of 1:2.

Source data are provided as a Source Data file.

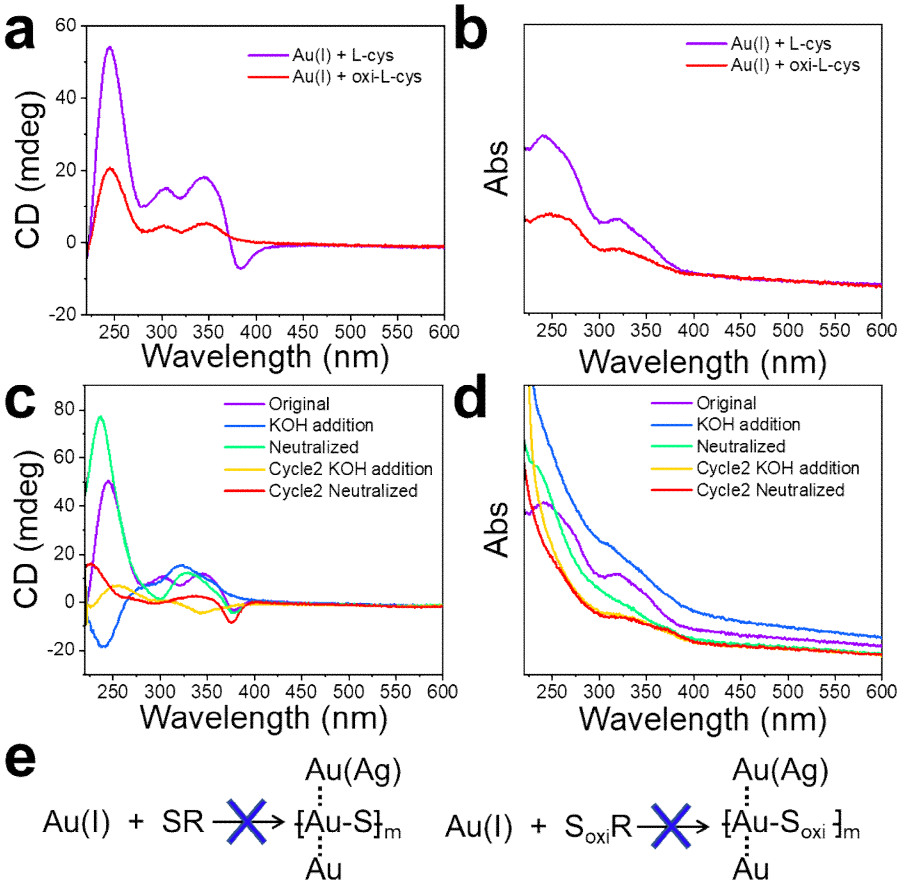

Process (1)

Supplementary Figure 32 Optical properties of the products obtained with L-cys and oxidized L-cys in an Au(I) to L-cys or oxi-L-cys ratio of 1:1.

(a, b) CD spectra and absorption spectra. For the reaction between Au(I) and L-cys, the Au(III) was reduced by 3 parts of AA first, and then 1 part of L-cys was added. For the reaction between Au(I) and oxidized L-cys, 1 part of L-cysteine was added into the $HAuCl_4$ solution to obtain oxidized L-cys, then 3 part of AA was added to fully reduce the Au(III) to Au(I). The responsive behavior of CP materials prepared with an Au(I) to L-cys ratio of 1:1 was further explored (c, d), and no chirality inversion behavior was found. Thus, it can be seen that Au(I) can react with L-cys or oxidized L-cys to form similar coordination polymers.

(c, d) responsive behavior of CP prepared by reacting Au(I). It can be seen that no chirality inversion behavior was found.

(e) Thus, CP1 cannot be obtained with a stoichiometry of 1:1 (e).

Source data are provided as a Source Data file.

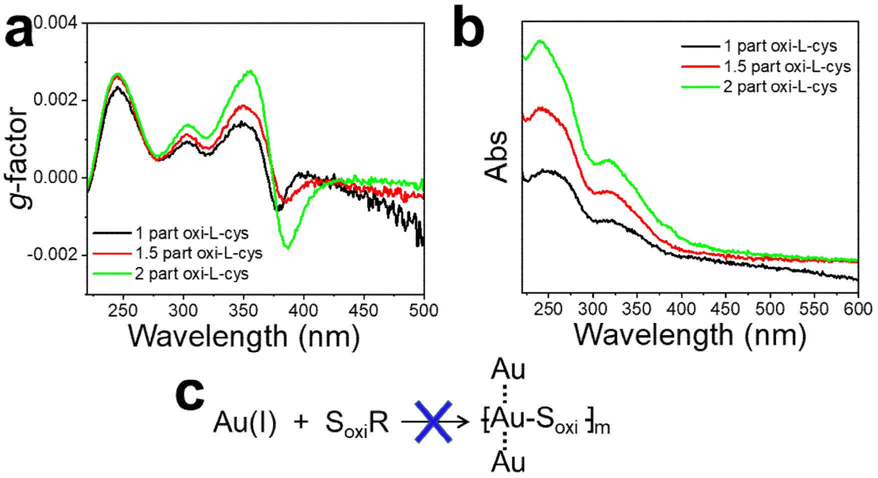

Supplementary Figure 33 Even if the amount of oxidized L-cysteine increased to 2 parts (the amount of $HAuCl_4$ was considered as 1 part) (green lines), no CP1 could be obtained.

(a) *g*-factor plots of CP materials prepared with different amount of oxidized L-cysteine; (b) UV-Vis spectra of CP materials prepared with different amount of oxidized L-cysteine. Note that the *g*-factor plots and UV-Vis spectra show distinct features with those of CP1. Therefore, the process in (c) will not occur.

Source data are provided as a Source Data file.

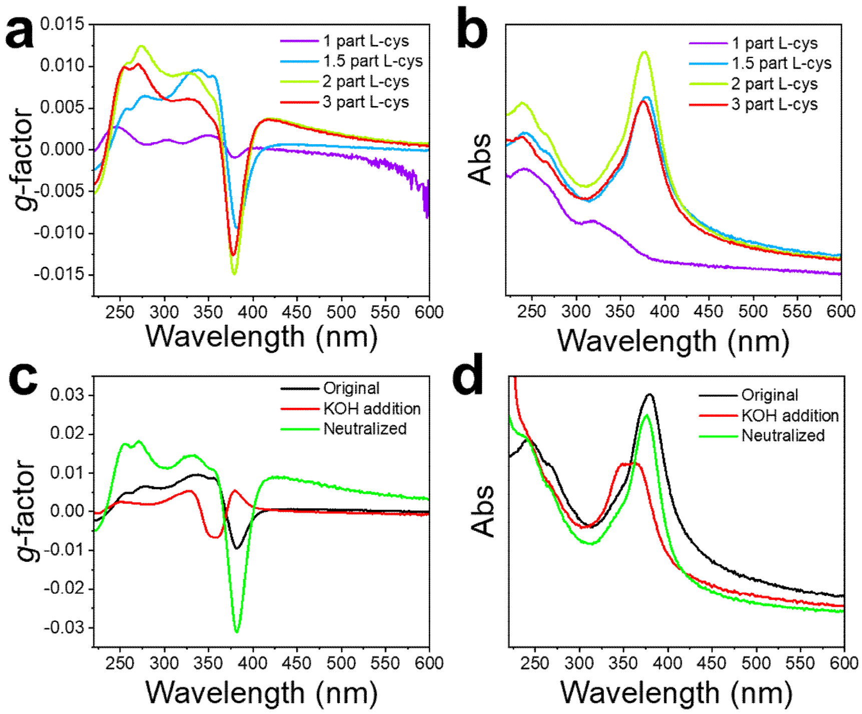

Supplementary Figure 34. The $HAuCl_4$ was reduced to Au(I) first via the reaction with AA, then different amounts of L-cysteine were added into the system, to test whether CP1 materials could be formed.

(a, b) *g*-factor plots and the UV-Vis spectra of the products obtained with different amounts of L-cysteine. When the amount of L-cysteine was increased to higher than 1.5 parts, CP materials with similar optical properties could be obtained.

(c, d) responsive behavior of the CP materials obtained with 1.5 part of L-cysteine in the synthesis. No chirality inversion was found.

Source data are provided as a Source Data file.

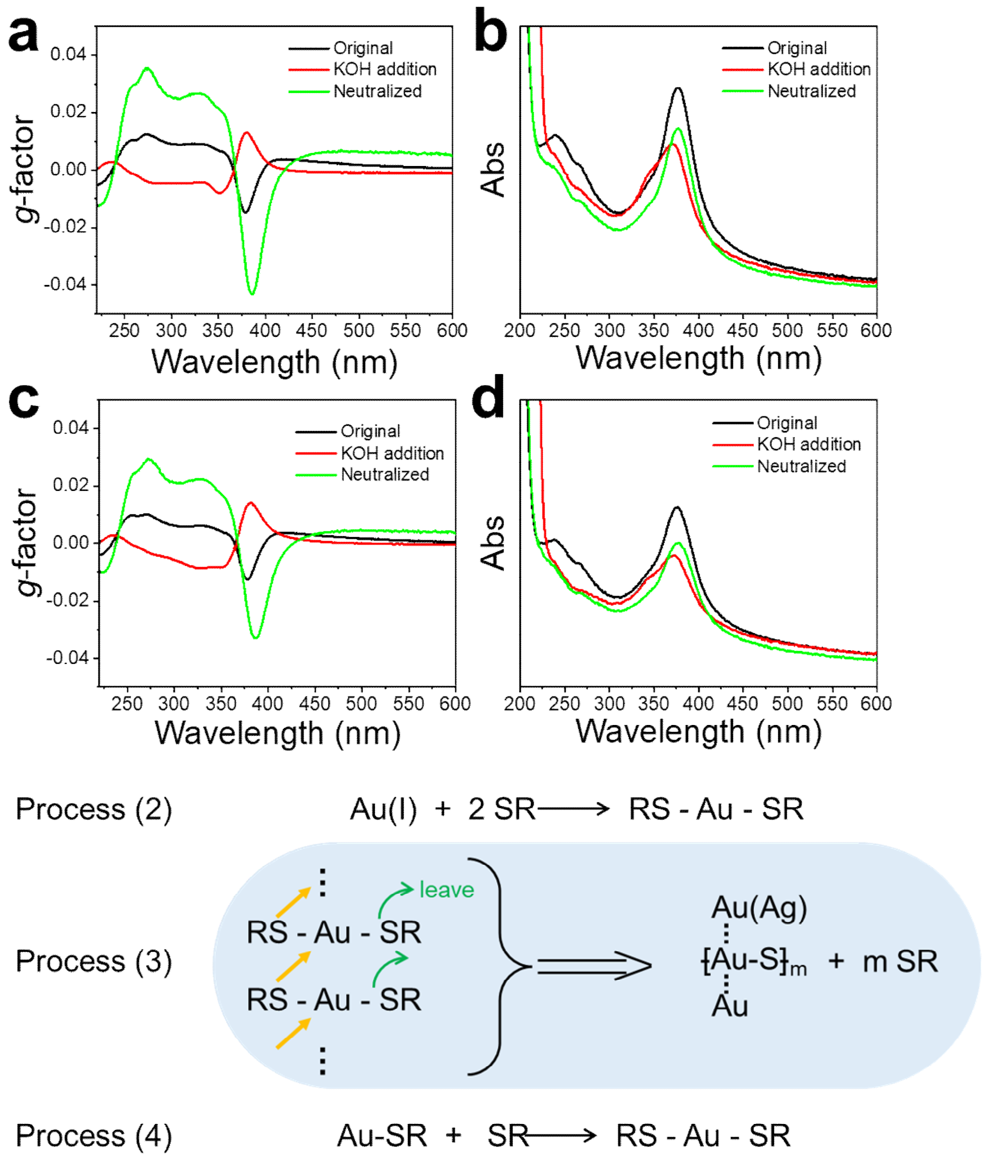

Supplementary Figure 35. Responsive behaviors of CP materials prepared with higher amount of L-cysteine. When the amount of L-cysteine was raised to 2 parts (a, b) or 3 parts (c, d), the obtained CP materials showed chirality inversion behavior. Thus, the obtained materials can be seen as CP1. Together with the results shown at Supplementary Figure 34, the formation of CP1 should involve the direction reaction between multiple RS-Au-SR species (Process (3)). Source data are provided as a Source Data file.

Supplementary Table 1. The atomic ratio of $AuAg_x$-cys coordination polymers determined by EDX

	Feeding Ag/Au	Ag	Au
L0	0	0	1
L0.06*	0.06	0.085	1
L0.2	0.2	0.20	1
L0.6	0.6	0.44	1
D0	0	0	1
D0.06	0.06	0.077	1
D0.2	0.2	0.19	1
D0.6	0.6	0.49	1
CTAC-L0.06	0.06	0.10	1
H2O-L0.2	0.2	0.28	1

* For L0.06, the Au:Ag ratio determined by XPS and ICP-OES were 1: 0.045 and 1:0.056, respectively.

Supplementary Table 2 Element percentage of L0 and L0.06 CP materials determined by XPS.

	Au	Ag	S	N	O	C
L0	5.72	n/a	5.63	7.32	46.88	34.46
L0.06	8.75	0.39	9.2	12.16	19.11	50.4

Supplementary References

1 Nie, H., Li, M., Hao, Y., Wang, X. & Zhang, S. X.-A. Time-resolved monitoring of dynamic self-assembly of Au (I)-thiolate coordination polymers. *Chem. Sci.* **4**, 1852-1857 (2013).

2 Fakhouri, H. *et al.* Sub-100 nanometer silver doped gold–cysteine supramolecular assemblies with enhanced nonlinear optical properties. *Physical Chemistry Chemical Physics* **21**, 12091-12099, doi:10.1039/C9CP00829B (2019).

3 Jiang, W. *et al.* Emergence of complexity in hierarchically organized chiral particles. *Science* **368**, 642-648, doi:doi:10.1126/science.aaz7949 (2020).

4 Russier-Antoine, I. *et al.* Chiral supramolecular gold-cysteine nanoparticles: Chiroptical and nonlinear optical properties. *Progress in Natural Science: Materials International* **26**, 455-460, doi:https://doi.org/10.1016/j.pnsc.2016.08.008 (2016).

5 Uvdal, K., Bodö, P. & Liedberg, B. l-cysteine adsorbed on gold and copper: An X-ray photoelectron spectroscopy study. *J. Colloid Interface Sci.* **149**, 162-173, doi:https://doi.org/10.1016/0021-9797(92)90401-7 (1992).

6 Kelemen, S. R., George, G. N. & Gorbaty, M. L. Direct determination and quantification of sulphur forms in heavy petroleum and coals: 1. The X-ray photoelectron spectroscopy (XPS) approach. *Fuel* **69**, 939-944, doi:https://doi.org/10.1016/0016-2361(90)90001-7 (1990).

7 Setiawan, L. D., Baumann, H. & Gribbin, D. Surface studies of keratin fibers and related model compounds using ESCA. I—intermediate oxidation products of the model compound 1-cystine and

their hydrolytical behaviour. *Surf. Interface Anal.* **7**, 188-195, doi:https://doi.org/10.1002/sia.740070406 (1985).

8 Yu, X.-R., Liu, F., Wang, Z.-Y. & Chen, Y. Auger parameters for sulfur-containing compounds using a mixed aluminum-silver excitation source. *J. Electron. Spectrosc. Relat. Phenom.* **50**, 159-166, doi:https://doi.org/10.1016/0368-2048(90)87059-W (1990).

9 Bain, C. D., Biebuyck, H. A. & Whitesides, G. M. Comparison of self-assembled monolayers on gold: coadsorption of thiols and disulfides. *Langmuir* **5**, 723-727, doi:10.1021/la00087a027 (1989).

10 Lindberg, B. J. *et al.* Molecular Spectroscopy by Means of ESCA II. Sulfur compounds. Correlation of electron binding energy with structure. *Phys. Scr.* **1**, 286, doi:10.1088/0031-8949/1/5-6/020 (1970).

11 Seah, M. P., Gilmore, I. S. & Beamson, G. XPS: binding energy calibration of electron spectrometers 5—re-evaluation of the reference energies. *Surf. Interface Anal.* **26**, 642-649, doi:https://doi.org/10.1002/(SICI)1096-9918(199808)26:9<642::AID-SIA408>3.0.CO;2-3 (1998).

12 Shen, J.-S. *et al.* Metal–Metal-Interaction-Facilitated Coordination Polymer as a Sensing Ensemble: A Case Study for Cysteine Sensing. *Langmuir* **27**, 481-486, doi:10.1021/la103153e (2011).

13 Deng, W.-F. *et al.* Inversion of Molecular Chirality Associated with Ferroelectric Switching in a High-Temperature Two-Dimensional Perovskite Ferroelectric. *J. Am. Chem. Soc.*, doi:10.1021/jacs.3c00634 (2023).
